# Clinical outcomes of left upper segmentectomy vs. lobectomy for early non-small-cell lung cancer: a nationwide database study in Japan

**DOI:** 10.1007/s00595-024-02844-8

**Published:** 2024-04-18

**Authors:** Shinya Tane, Jiro Okami, Yoshimasa Maniwa, Yasushi Shintani, Hiroyuki Ito, Takashi Ohtsuka, Shinichi Toyooka, Takeshi Mori, Shun-ichi Watanabe, Masayuki Chida, Shunsuke Endo, Ryoichi Nakanishi, Mitsutaka Kadokura, Hidemi Suzuki, Etsuo Miyaoka, Ichiro Yoshino, Hiroshi Date, Japanese Joint Committee of Lung Cancer Registry

**Affiliations:** 1 Division of Thoracic Surgery, Department of Surgery, Kobe University Graduate School of Medicine, Kobe, Japan; 2 Department of General Thoracic Surgery, Osaka International Cancer Institute, Osaka, Japan; 3 Department of General Thoracic Surgery, Osaka University Graduate School of Medicine, Suita, Japan; 4 Department of Thoracic Surgery, Kanagawa Cancer Center, Yokohama, Japan; 5 Division of General Thoracic Surgery, Department of Surgery, Jikei University School of Medicine, Tokyo, Japan; 6 Department of Thoracic, Breast and Endocrinological Surgery, Graduate School of Medicine, Dentistry and Pharmaceutical Sciences, Okayama University, Okayama, Japan; 7 Department of Thoracic Surgery, Japanese Red Cross Kumamoto Hospital, Kumamoto, Japan; 8 Department of Thoracic Surgery, National Cancer Center Hospital, Tokyo, Japan; 9 Department of General Thoracic Surgery, Dokkyo Medical University, Mibu, Japan; 10 Department of Thoracic Surgery, Jichi Ika University Saitama Medical Center, Saitama, Japan; 11 Department of Oncology, Immunology and Surgery, Nagoya City University Graduate School of Medical Sciences, Nagoya, Japan; 12 Respiratory Disease Center, Showa University Northern Yokohama Hospital, Yokohama, Japan; 13 Department of General Thoracic Surgery, Graduate School of Medicine, Chiba University, Chiba, Japan; 14 Department of Mathematics, Tokyo University of Science, Tokyo, Japan; 15 Department of Thoracic Surgery, School of Medicine, International University of Health and Welfare, Narita, Japan; 16 Department of Thoracic Surgery, Kyoto University Graduate School of Medicine, Kyoto, Japan

**Keywords:** Left upper lobe, Non-small-cell lung cancer, Prognosis, Segmentectomy

## Abstract

**Purpose:**

Given that left upper lobe and right upper and middle lobes share a similar anatomy, segmentectomy, such as upper division and lingulectomy, should yield identical oncological clearance to left upper lobectomy. We compared the prognosis of segmentectomy with that of lobectomy for early stage non-small-cell lung cancer (NSCLC) in the left upper lobe.

**Methods:**

We retrospectively examined 2115 patients who underwent segmentectomy or lobectomy for c-stage I (TNM 8th edition) NSCLC in the left upper lobe in 2010. We compared the oncological outcomes of segmentectomy (*n* = 483) and lobectomy (*n* = 483) using a propensity score matching analysis.

**Results:**

The 5-year recurrence-free and overall survival rates in the segmentectomy and lobectomy groups were comparable, irrespective of c-stage IA or IB. Subset analyses according to radiological tumor findings showed that segmentectomy yielded oncological outcomes comparable to those of lobectomy for non-pure solid tumors. In cases where the solid tumor exceeded 20 mm, segmentectomy showed a recurrence-free survival inferior to that of lobectomy (*p* = 0.028), despite an equivalent overall survival (*p* = 0.38).

**Conclusion:**

Segmentectomy may be an acceptable alternative to lobectomy with regard to the overall survival of patients with c-stage I NSCLC in the left upper lobe.

## Introduction

The role of segmentectomy has expanded to include resection of peripheral small-sized early non-small-cell lung cancer (NSCLC), as this technique may better preserve the lung function than lobectomy [1, 2]. Recently, a Japanese randomized controlled trial to confirm the non-inferiority of segmentectomy to lobectomy in patients with small peripheral NSCLC (JCOG0802/WJOG4607L) revealed a significantly better overall survival (OS) with segmentectomy than lobectomy, suggesting that it could become the standard treatment instead of lobectomy for this population [3]. Although segmentectomy exhibits a significantly higher rate of local relapse than lobectomy [4], this result may help us perform less-invasive resection of a smaller volume of lung tissue and facilitates tailor-made surgery for each patient.

As the left upper lobe is one of the largest lobes in the lungs, segmentectomies, such as upper division segmentectomy and lingulectomy, are well established as acceptable treatments in thoracic surgery. In particular, the anatomy of the left upper division (segments 1+2 and 3) and lingula (segments 4 and 5) is similar to that of the right upper and middle lobes. Several studies have demonstrated equal oncological outcomes between split lobectomy (upper division segmentectomy or lingulectomy) and left upper lobectomy [5–9]. We previously compared the oncological outcomes between upper division segmentectomy and lobectomy in the left upper division located in cN0 NSCLC and demonstrated that oncological outcomes following upper division segmentectomy were not inferior to those following lobectomy, even when the tumor was larger than 2 cm and located close to the intersegmental plane [10]. However, all previous studies were reported from single institutions, with no large multi-institutional cohort studies conducted to date.

The 7th Japanese Joint Committee of Lung Cancer Registry (JJCLCR) is one of the most extensive nationwide databases, having collected information on clinicopathological factors from 18,973 patients at 297 hospitals who underwent pulmonary resection for lung cancer in 2010 [11]. Using this database, we reassessed the impact and feasibility of left upper segmentectomy in comparison with left upper lobectomy for c-stage I NSCLC located in the left upper lobe, based on oncological outcomes.

## Patients and methods

### Ethics statement

The study was approved by the Review Board of Osaka University Hospital (approval no. 15321) following the ethical guidelines for epidemiologic studies, and the need to obtain written informed consent from the patients was waived.

### Patients

Patients with c-stage I NSCLC (TNM 8th edition) in the left upper lobe who underwent either left upper lobectomy or segmentectomy in 2010 were enrolled in this study. Although all the data were registered based on the TNM 7th edition, the data regarding c-stage were reviewed based on the TNM 8th edition. The exclusion criteria were as follows: (1) history of thoracic surgery or lung cancer; (2) history of other cancers within five years (3) induction therapy; and (4) combined resection of other adjacent organs, bronchoscopy, or incomplete resection.

### Outcomes

Postoperative recurrence was recorded based on the physician’s diagnosis at the institution. The OS was defined as the time from the date of surgery to the date of death from any cause. The recurrence-free survival (RFS) was defined as the time from the date of surgery to the date of local or distant recurrence or death.

### Statistical analyses

Descriptive statistics included mean and standard deviation ranges for continuous variables and percentages for categorical variables. The Mann–Whitney U test was used to compare continuous variables, and Fisher’s exact test was used to compare nominal variables. The OS and RFS curves were drawn according to the Kaplan–Meier method, and differences were tested using the log-rank method.

In addition, we conducted a propensity score matching (PSM) analysis to minimize potential data bias using a ratio and caliper distance of 1:1 and 0.05, respectively.

Patients with unreported data were excluded from the analysis due to missing data. Statistical significance was set at *p* < 0.05.

## Results

### Oncological outcomes according to the clinical stage in the left upper lobe

A total of 2115 patients who underwent lobectomy or segmentectomy for c-stage I (TNM 8th edition) NSCLC located in the left upper lobe were included in the current study. Among them, 557 patients underwent segmentectomy, and 1558 underwent lobectomy. The demographic and oncological characteristics of patients are shown in Table 1. Age, sex distribution, performance status (PS), smoking history, presence of emphysema and fibrosis, and tumor histology were similar between the groups. Patients who underwent segmentectomy showed a lower pulmonary function, lower serum levels of carcinoembryonic antigen (CEA), lesser extent of lymph node dissection, lower whole and invasive tumor sizes, a better pathological stage, a lower rate of receiving adjuvant therapy, and a lower rate of recurrence than those who underwent lobectomy.

**Table 1:** Surgical and oncological outcomes before propensity score matching

	Segmentectomy (*n* = 557)	Lobectomy (*n* = 1558)	*p* value
Age (<65 vs. ≥65 years)	163/394	522/1036	0.073
Sex (M vs. F)	303/254	913/645	0.090
ECOG PS			0.145
0	466	1367	
1 or higher	91	184	
FEV1.0 (L)	2.17 ± 0.61	2.27 ± 0.62	0.002
FEV1.0/FVC (%)	73.2 ± 10.5	74.2 ± 9.5	0.048
Smoking history (yes)	296 (5)	886 (59.9)	0.136
Emphysema on CT	79 (14.2)	177 (11.4)	0.082
Fibrosis on CT	20 (3.6)	34 (2.2)	0.084
Serum level of CEA >5	100 (18.0)	417 (26.8)	<0.001
Lymph node dissection			<0.001
ND0	62	10	
ND1	260	233	
ND2	225	1303	
Unknown	10	12	
Operation time (min)	210 ± 78	213 ± 72	0.376
Overall postoperative complications (%)	35(6.3)	107(6.9)	0.694
Prolonged air leak (%)	17 (3.0)	43 (2.7)	
Histology			0.190
Adenocarcinoma	451	1220	
Squamous cell carcinoma	78	226	
Others or unknown	28	112	
Whole tumor size (mm)	1.9 ± 0.8	2.4 ± 1.0	<0.001
Invasive tumor size (mm)	1.3 ± 0.9	2,1 ± 1.0	<0.001
The location of tumor (upper division/lingula)	432/125	1228/270	<0.001
Clinical stage (UICC ver.8)			<0.001
0	82	49	
IA1	177	184	
IA2	176	510	
IA3	79	470	
IB	43	345	
Pathological T (UICC ver.7)			<0.001
Tx	1	2	
T1a	355	500	
T1b	107	440	
T2a	86	553	
T2b	3	19	
T3	5	43	
T4	0	1	
Pathological N (UICC ver.7)			<0.001
NX	24	33	
N0	514	1824	
N1	10	117	
N2	9	141	
Pathological stage (UICC ver.7)			<0.001
0 or X	26	11	
IA	431	842	
IB	75	425	
IIA	11	115	
IIB	5	28	
IIIA	9	137	
Adjuvant chemotherapy (%)	50 (9.0)	468 (30.0)	<0.001
Recurrence (%)	59 (10.6)	400 (21.9)	<0.001
Regional	37 (6.6)	150 (9.6)	0.037
Distance	26 (4.7)	218 (14.0)	<0.001

*CEA* carcinoembryonic antigen, *CT* computed tomography, *ECOG PS* Eastern Cooperative Oncology Group performance status, *FEV1* forced expiratory volume in 1 s, *FVC* forced vital capacity

### Comparisons between segmentectomy and lobectomy in the left upper lobe after PSM

**Fig. 1:**
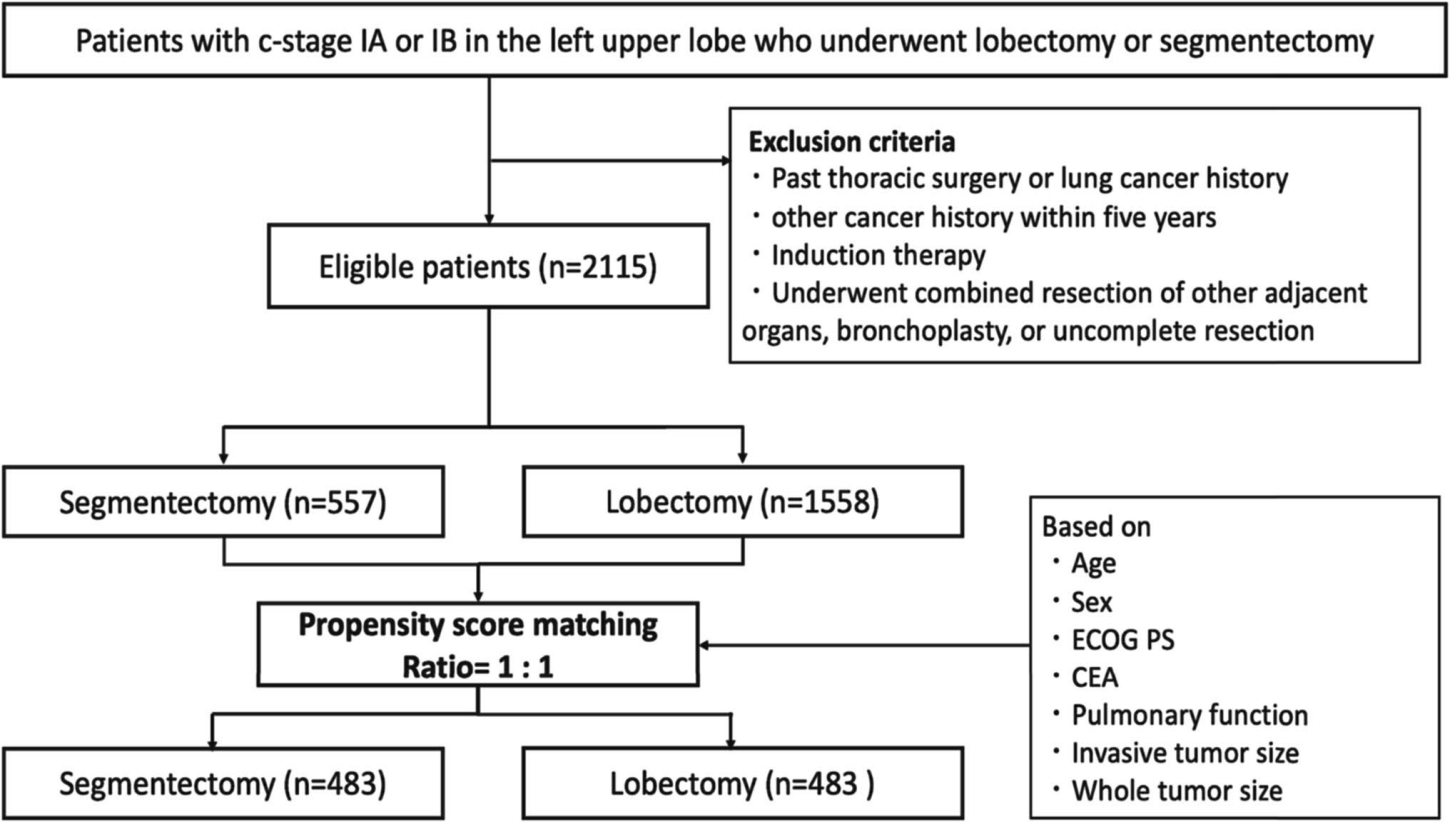
Flowchart of study population selection

To reduce selection bias, 483 patients who underwent segmentectomy were matched with 483 patients who underwent lobectomy, based on their age, sex, PS, CEA, pulmonary function, and whole and invasive tumor sizes (Fig. 1). The demographic and oncological characteristics of patients post-PSM matching are summarized in Table 2. Even after matching, the patients who underwent segmentectomy exhibited a lesser extent of lymph node dissection, better pathological stage, and a lower rate of receiving adjuvant therapy than those who underwent lobectomy. Overall postoperative complications, including prolonged air leak, did not remarkably vary between the segmentectomy and lobectomy groups (6.2 vs. 8.9%, *p* = 0.14). In addition, the operative duration was not significantly different between the segmentectomy and lobectomy groups (211 vs. 214 min, *p* = 0.53).

**Table 2:** Surgical and oncological outcomes after propensity score matching

	Segmentectomy (*n* = 438)	Lobectomy (*n* = 438)	*p* value
Age (<65 vs. ≥65 years)	145/338	139/344	0.72
Sex	263/220	269/214	0.75
ECOG PS			0.75
0	406	412	
1 or higher	77	71	
FEV1.0 (L)	2.19 ± 0.63	2.16 ± 0.61	0.95
FEV1.0/FVC (L)	73.3 ± 10.6	72.6 ± 10.1	0.28
Smoking history (Yes/No)	265/218	268/215	0.897
Emphysema on CT	71 (14.7)	55 (11.4)	0.15
Fibrosis on CT	18 (3.7)	10 (2.1)	0.18
Serum level of CEA (> 5)	96 (19.9)	112 (23.2)	0.24
Lymph node dissection			<0.001
ND0	54	5	
ND1	223	89	
ND2	197	385	
Unknown	9	4	
Operation time (min)	211 ± 80	214 ± 72	0.53
Overall postoperative complications (%)	30 (6.2)	43 (8.9)	0.14
Prolonged air leak (%)	14 (2.9)	16 (3.3)	0.85
Histology			0.37
Adenocarcinoma	380	389	
Squamous cell carcinoma	75	61	
Others or unknown	28	33	
Whole tumor size (mm)	1.9 ± 0.8	2.0 ± 0.8	0.10
Invasive tumor size (mm)	1.4 ± 0.9	1.4 ± 0.9	0.78
The location of tumor (upper division/lingula)	373/110	408/75	0.003
Clinical stage (UICC ver.8)			0.396
0	51	41	
IA1	145	133	
IA2	166	194	
IA3	79	78	
IB	42	37	
Pathological T (UICC ver.7)			0.158
T1a	295	264	
T1b	99	101	
T2a	83	107	
T2b	1	3	
T3	5	8	
T4	0	0	
Pathological N (UICC ver.7)			<0.001
Nx	19	4	
N0	446	433	
N1	9	18	
N2	9	28	
Pathological stage (UICC ver.7)			<0.001
0orX	20	4	
IA	369	341	
IB	72	86	
IIA	8	19	
IIB	5	5	
IIIA	9	28	
Adjuvant chemotherapy (%)	48 (10.7)	92 (20.3)	<0.001
Recurrence (%)	56 (11.6)	77 (15.9)	0.062
Regional	35 (7.2)	42 (8.7)	0.48
Distance	25 (5.2)	46 (9.3)	0.018

*CEA* carcinoembryonic antigen, *CT* computed tomography, *ECOG PS* Eastern Cooperative Oncology Group performance status, *FEV1* forced expiratory volume in 1 s, *FVC* forced vital capacity

**Fig. 2:**
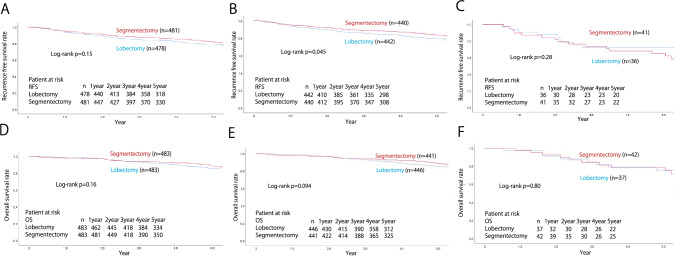
The recurrence-free and the overall survival of the overall cohort (**A**, **D**), c-stage IA (**B**, **E**), and c-stage IB (**C**, **F**) of the matched cohort

**Fig. 3:**
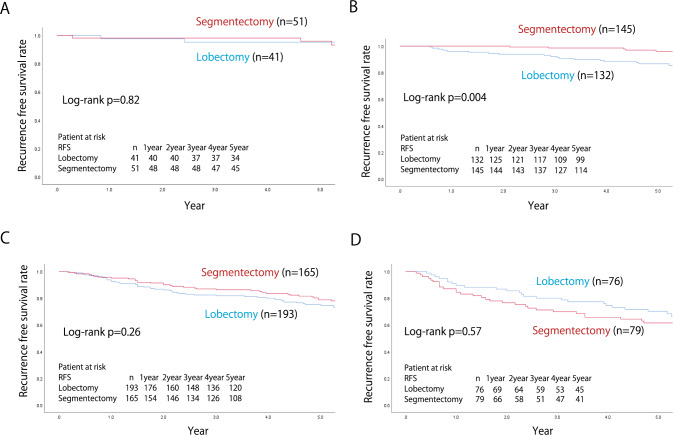
The recurrence-free survival of c-stage 0 (**A**), c-stage IA1 (**B**), c-stage IA2 (**C**) and c-stage IA3 (**D**) cases in the matched cohort

**Fig. 4:**
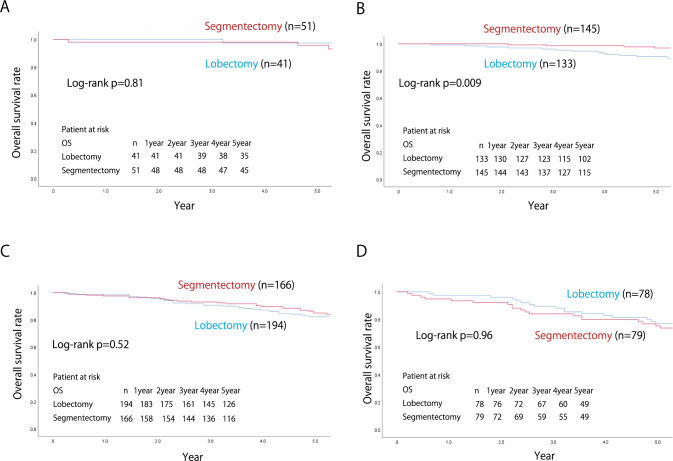
The overall survival of c-stage 0 (**A**), c-stage IA1 (**B**), c-stage IA2 (**C**), and c-stage IA3 (**D**) cases in the matched cohort

The median follow-up time was 66.5 ± 0.29 months, calculated using the reverse Kaplan–Meier curve [12]. A comparison of the 5-year RFS and OS rates between segmentectomy and lobectomy cases in each group is shown in Fig. 2. The respective 5-year RFS and OS rates in the segmentectomy and lobectomy groups were 81.9 vs. 78.9% and 87.2 vs. 84.6%, respectively, and the differences between the procedures were not significant (*p* = 0.15 and *p* = 0.16, respectively). When stratified by c-stage (UICC ver.8), the respective 5-year RFS and OS rates in each procedure were 84.2 vs. 80.6% (*p* = 0.082) and 88.3 vs. 85.8% (*p* = 0.14) in c-stage 0 or IA, and 66.6 vs. 67.2% (*p* = 0.87) and 79.8 vs. 76.3% (*p* = 0.80) in stage IB, demonstrating that outcomes between segmentectomy and lobectomy were comparable, irrespective of c-stage. Patients with c-stage IA were divided into c-stage0, c-stage IA1, c-stage IA2, and c-stage IA3, and the 5-year RFS and OS rates were compared for each procedure. The respective 5-year RFS and OS rates in segmentectomy and lobectomy were 95.9 vs. 95.1% (*p* = 0.82), and 95.9 vs. 97.4% (*p* = 0.81) in c-stage 0, and 96.1 vs. 86.6% (*p* = 0.004) and 97.0 vs. 90.5% (*p* = 0.009) in c-stage IA1, 78.7 vs. 74.6% (*p* = 0.26) and 85.0 vs. 82.3% (*p* = 0.52) in stage IA2, 84.4 vs. 89.5% (*p* = 0.57) and 73.7 vs. 76.9% (*p* = 0.96) in stage IA3 (Figs. 3 and 4).

The type of recurrence and the incidence of regional and distant recurrences after each procedure are displayed in Table 2. During the follow-up, 56 patients developed recurrence following segmentectomy (11.6%), and 77 developed recurrence following lobectomy (15.9%). Regional recurrence occurred in 35 patients (7.2%) who underwent segmentectomy and 42 patients (8.7%) who underwent lobectomy. Distant metastasis was reported in 25 (5.2%) and 46 (9.3%) patients in the segmentectomy and lobectomy groups, respectively.

### Comparison based on radiological findings [ground-glass opacity (GGO)-dominant, solid-dominant, and pure solid tumor] in tumors ≤20 or >20 mm in diameter

Subsequently, we assessed the impact of each procedure on oncological outcomes according to radiological tumor findings. The tumors were classified by the consolidation/tumor ratio (CTR) and divided into 3 groups: GGO-dominant tumor (CTR ≤ 0.5), solid-dominant tumor (CTR > 0.5 to < 1), and pure solid tumor (CTR = 1).

**Fig. 5:**
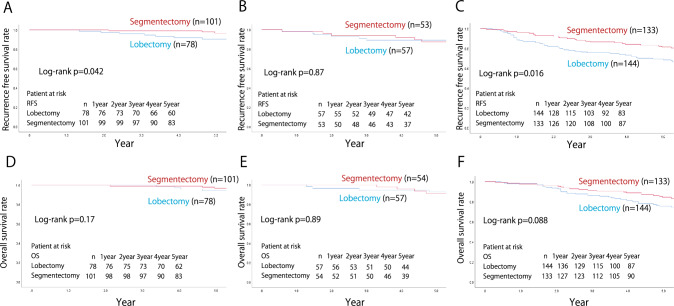
The recurrence-free and the overall survival of cases with a tumor diameter ≤ 20 mm corresponding to the following radiological findings: **A**, **D** GGO-dominant tumor (CTR ≤ 0.5), **B**, **E** solid-dominant tumor (CTR > 0.5 and < 1), and **C**, **F** pure solid tumor (CTR = 1). GGO, ground-glass opacity

As shown in Fig. 5, in cases where the whole tumor diameter was ≤ 20 mm with GGO- or solid-dominant features, the 5-year RFS and OS rates did not vary markedly between segmentectomy and lobectomy [GGO-dominant: 96.7 vs. 90.4% (*p* = 0.42) and 96.7 vs. 94.4% (*p* = 0.17), solid-dominant: 87.4 vs. 89.1% (*p* = 0.88) and 91.4 vs. 92.6% (*p* = 0.89)]. Regarding pure solid tumors, segmentectomy showed a better RFS than lobectomy (81.5 vs. 68.5%; *p* = 0.016). However, the OS was not significantly different between segmentectomy and lobectomy (84.4 vs. 75.6%; *p* = 0.088).

**Fig. 6:**
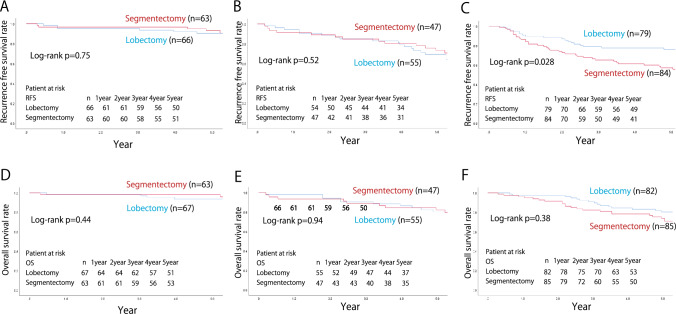
The recurrence-free and the overall survival with tumor diameter > 20 mm corresponding to the following radiological findings: **A**, **D** GGO-dominant tumor (CTR ≤ 0.5), **B**, **E** solid-dominant tumor (CTR > 0.5 and < 1), and **C**, **F** pure solid tumor (CTR = 1). GGO, ground-glass opacity

The aforementioned trend followed the same trend as that of the whole tumor diameter of >20 mm. Even in cases where the whole tumor diameter was >20 mm with GGO- or solid-dominant features, the 5-year RFS and OS rates did not differ markedly between segmentectomy and lobectomy [GGO-dominant; 93.2 vs. 90.5% (*p* = 0.75) and 98.4 vs. 93.7% (*p* = 0.44), solid-dominant; 73.3 vs. 69.3% (*p* = 0.52) and 82.3 vs. 80.3% (*p* = 0.94)] (Fig. 6). However, for pure solid tumors, the RFS of segmentectomy was worse outcomes than that of lobectomy (57.2 vs. 76.1%; *p* = 0.028), while the OS was not significantly different between segmentectomy and lobectomy (70.9 vs. 80.3%; *p* = 0.38). Regarding the type of recurrence for pure solid tumors >20 mm, regional recurrence occurred in 16 patients (18.8%) who underwent segmentectomy and 8 patients 9.8%) who underwent lobectomy (*p* = 0.092). Distant metastasis was reported in 15 (17.7%) and 14 (17.0%) patients in the segmentectomy and lobectomy groups, respectively (*p* = 0.92).

## Discussion

Recently, multi-institutional randomized clinical trials (JCOG0802/WJOG4607L and CALGB140503) demonstrated the clinical value of segmentectomy in NSCLC. However, this benefit is limited to patients with small-sized peripheral NSCLC [3, 13]. Given the similar anatomical classification of the left upper division and lingula to that of the right upper and middle lobes, those who underwent segmentectomy (i.e. upper division segmentectomy and lingulectomy) in the left upper lobe may benefit in terms of the OS, even for tumors larger than 20 mm in diameter.

Several studies have reported similar oncological outcomes between left upper segmentectomy (upper division segmentectomy and lingulectomy) and left upper lobectomy. Among these studies, one of the largest was conducted by Zhou et al. who performed PSM, resulting in 273 pairs of patients undergoing thoracoscopic left upper division segmentectomy or left upper lobectomy for stage I NSCLC. The authors reported no compelling differences in clinical or oncological outcomes between the groups [9]. Furthermore, our institution also reported that the clinical and oncological outcomes following left upper division segmentectomy for clinical N0 NSCLC were not significantly inferior to those following lobectomy, even if the tumor was located close to the intersegmental plane as measured by three-dimensional computed tomography [10]. Although some studies have demonstrated similar oncological outcomes for left upper segmentectomy and lobectomy (5–9), our study cohort also included patients who underwent other single segmentectomy procedures, such as S1+2 segmentectomy. Since we assumed that all these segmentectomies for the left upper lobe were performed as a ‘split-lobe’ procedure, further studies are required to clarify whether or not the oncological clearance of this split-lobe concept would be applicable for all forms of segmentectomy in the left upper lobe. To our knowledge, this multi-institutional study has the largest cohort showing the non-inferiority of left upper segmentectomy to left upper lobectomy.

Corresponding to previous studies, this study revealed that segmentectomy exhibited an OS comparable to that of left upper lobectomy, irrespective of tumor size. However, the differences between the two groups in terms of pathological characteristics were concerning. Despite adjusting for the characteristics of segmentectomy and lobectomy, including tumor size, the rate of pN1/2 was significantly higher in the lobectomy group than in the segmentectomy group. One reason is that lobe-specific lymph node dissection may have been omitted during segmentectomy. For instance, although complete interlobar lymph node dissection is challenging, owing to variations in the divergence style of the lingular artery and vein during upper division segmentectomy, NSCLC located in the upper division tends to metastasize to the interlobar lymph nodes [14]. As presented in Table 2, mediastinal lymph node dissection during segmentectomy was not performed in some cases, which may have led to underestimation of the pathological stage in the segmentectomy group. Nevertheless, segmentectomy revealed an OS comparable to that of lobectomy. Indeed, a recent study also suggested that the survival was similar between lobectomy and segmentectomy in patients with clinical N0 and unsuspected pathological N1/N2 nodal metastases [15].

One of the most notable concerns after segmentectomy is local recurrence in the residual lobe [16]. Therefore, sufficient tumor margins should be ensured. Theoretically, as the subpleural lymphatic pathway can be blocked by the intersegmental septum, accurate intersegmental dissection during segmentectomy may allow resection of the tumor in the affected segment without the infiltration of cancer cells into the neighboring segment. As margins equal to the tumor diameter or >20 mm would be acceptable [17], and pleural lymphatic drainage might follow an intersegmental pathway [18], dissection into the neighboring segment while sacrificing the intersegmental vein is necessary if the tumor is close to the intersegmental plane. In addition, if the tumor located in the right upper or middle lobe is close to the minor fissure, most thoracic surgeons prefer to spare the middle or upper lobe and dissect into the neighboring lobe to secure the margin instead of performing bi-lobectomy. This may be applicable for large tumors (>20 mm) located in the left upper lobe during split-lobe segmentectomy if the tumor margin is sufficiently secured.

Early-stage NSCLC with a GGO component has been reported to be a uniform group of tumors that exhibit low-grade malignancy and have an excellent prognosis [19]. In contrast, solid predominant or pure solid tumors have more malignant potential, such as lymph node metastasis [20]. Our study revealed that left upper segmentectomy for radiological GGO or solid-dominant stage I NSCLC had long-term effects similar to those of left upper lobectomy, even when the entire tumor size exceeded 20 mm. Interestingly, segmentectomy had a significant advantage with regard to the RFS in patients with solid tumors ≤20 mm in size. One possible reason for this is that pathological upstaging might be infrequent in segmentectomy, probably because segmentectomy may be likely to be applied to cases with peripherally located tumors, which are less strongly associated with lymph node metastasis than inner-located tumor [21]. In contrast, segmentectomy showed a significantly worse RFS than lobectomy for solid tumors >20 mm, as segmentectomy might carry a high risk of recurrence due to insufficient hilar lymph node dissection. Nonetheless, segmentectomy exhibited an OS equivalent to that of lobectomy. One possible reason for this is that segmentectomy, which preserves more of the lung parenchyma than lobectomy, might have allowed more extensive treatment for relapse of primary lung cancer and second primary lung cancer than lobectomy, despite its higher recurrence rate. Thus, segmentectomy offers a prognosis similar to that of lobectomy, even for pure solid tumors.

Several limitations associated with the present study warrant mention. First, despite the propensity matching analysis, selection bias for surgical procedures and other biases may still exist. Indeed, thoracic surgeons performed segmentectomy as curative-intent resection for patients with predominantly GGO, low metabolic activity, slow-growth NSCLC, and compromised limited resection for patients unable to tolerate lobectomy. Second, this dataset, which was based on surgical cases in 2010, lacked information on minimally invasive surgical approaches, such as uniportal-, multiportal-, and robot-assisted thoracoscopic surgery. Third, our dataset lacked information regarding tumor location (i.e. tumor centrality and the specific segment location) and pathological margins. Finally, as the current study was retrospective, further multi-institutional prospective randomized trials are warranted in the future.

In conclusion, the current analyses suggest that the OS following segmentectomy for c-stage I NSCLC in the left upper lobe is not significantly inferior to that following lobectomy, irrespective of the tumor size and radiological tumor findings.
